# Enhancing Multicenter Trials With the Trial Innovation Network's Initial Consultation Process

**DOI:** 10.1001/jamanetworkopen.2025.12926

**Published:** 2025-05-29

**Authors:** Paul A. Harris, Consuelo H. Wilkins, Karen Lane, Gordon R. Bernard, Jonathan D. Casey, Daniel E. Ford, Salina P. Waddy, Ken L. Wiley, Terri L. Edwards, Nichol McBee, Dixie D. Thompson, Mary Stroud, Emily Serdoz, Nan Kennedy, Sarah J. Nelson, Michelle Jones, Lindsay M. Eyzaguirre, Leslie R. Boone, Jessica Baird, Colleen Lawrence, Elizabeth Holthouse, Sarah K. Cook, Maeve Tischbein, Natalya Amrine, Tiffany Chen, Jodie Cohen, LaShondra Deyampert, Natalie Dilts, Delicia Burts, Amna Baig, Joseph Christodoulou, Mariela Rodriguez, Edgar R. Miller, James F. Casella, W. Andrew Mould, J. Michael Dean, Daniel K. Benjamin, Harry P. Selker, Marisha E. Palm, Lori Poole, Jeri S. Burr, Sara Hassani, Angeline Nanni, Meghan Hildreth, Daniel F. Hanley

**Affiliations:** 1 Vanderbilt Institute for Clinical and Translational Research, Nashville, Tennessee; 2 Department of Biomedical Informatics, Vanderbilt University Medical Center, Nashville, Tennessee; 3 Department of Medicine, Vanderbilt University Medical Center, Nashville, Tennessee; 4 Department of Internal Medicine, Meharry Medical College, Nashville, Tennessee; 5 Johns Hopkins University School of Medicine, Baltimore, Maryland; 6 Duke Clinical Research Institute, Durham, North Carolina; 7 Johns Hopkins Institute for Clinical and Translational Research, Baltimore, Maryland; 8 National Center for Advancing Translational Sciences, Bethesda, Maryland; 9 University of Utah Health, Salt Lake City; 10 Utah Clinical and Translational Science Institute, Salt Lake City; 11 Duke University School of Medicine, Durham, North Carolina; 12 Institute for Clinical Research and Health Policy Studies, Tufts Medical Center, Boston, Massachusetts; 13 Tufts Clinical and Translational Science Institute, Tufts University, Boston, Massachusetts

## Abstract

This qualitative study describes the Trial Innovation Network's initial consultation process, which provides researchers with resources and recommendations to address complex aspects of planning and conducting clinical trials.

## Introduction

The National Institutes of Health’s National Center for Advancing Translational Sciences established the Trial Innovation Network (TIN) as national infrastructure to address multicenter trial barriers and offer investigators access to a scientific consultative process, clinical trial and disease experts, and methods across the trial life cycle.^1,2^ The TIN initial consultation process provides researchers with resources and recommendations to address complex aspects of planning and conducting more informative clinical trials; data-driven solutions for site identification, representative recruitment, and retention planning; data management; and regulatory compliance.

## Methods

This qualitative study reports on TIN Initial Consultations from proposals submitted to the TIN from October 26, 2016, until June 1, 2024. This study followed the Standards for Reporting Qualitative Research (SRQR) reporting guideline and did not involve human participants research; therefore, as set out in the Federal Policy for the Protection of Human Subjects, codified at 45 CFR 46.102, Institutional review board (IRB) approval was not required..

**Figure 1.**
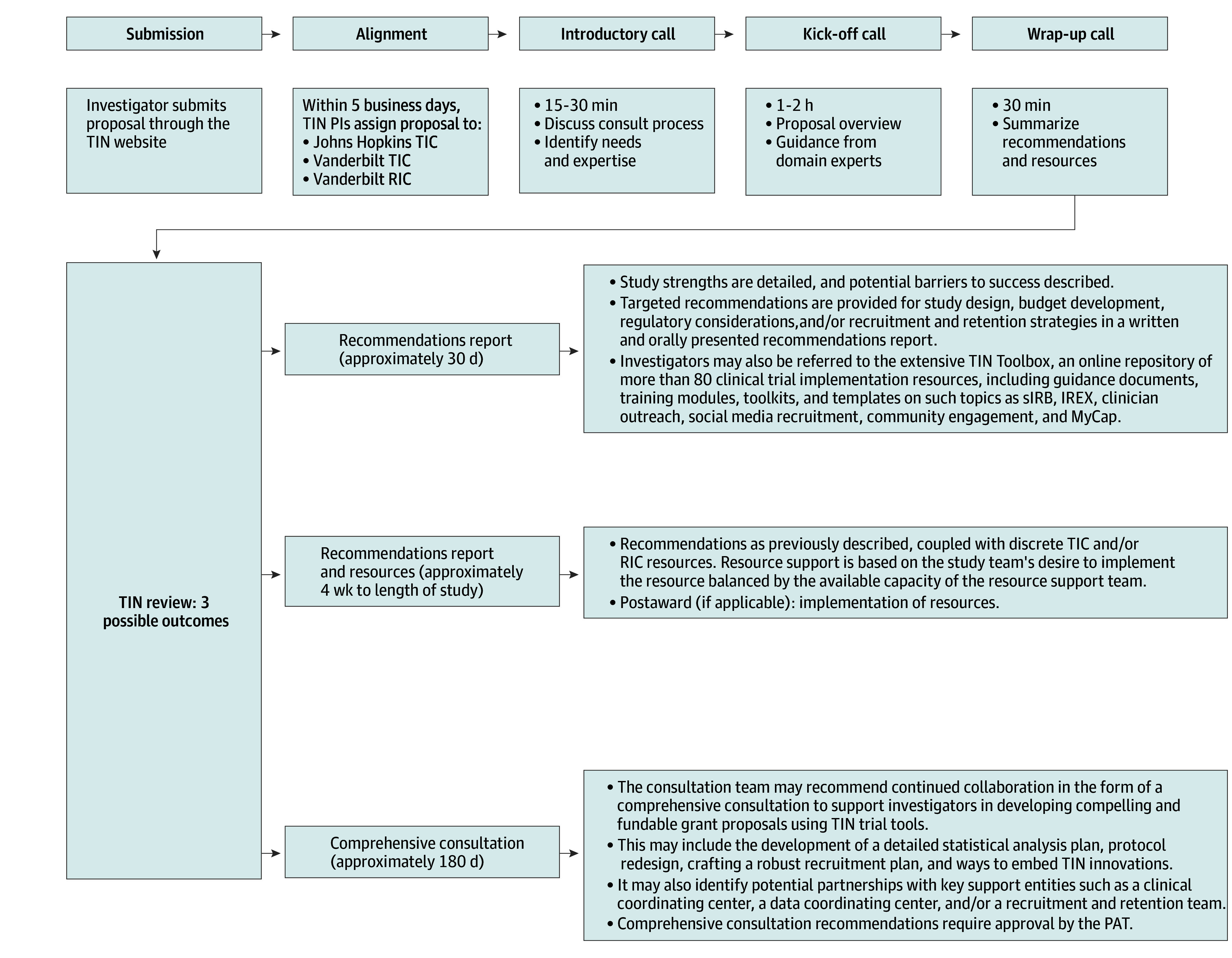
Schematic of Initial Consultation Process PAT, Proposal Assessment Team; PI, principal investigator; RIC, Recruitment Innovation Center; TIC, Trial Innovation Center; TIN, Trial Innovation Network.

A proposal requesting a TIN consultation for a planned or current multicenter study is submitted by the study investigator via the TIN’s website portal. The proposal is reviewed within 5 days, aligned with a Trial Innovation Center (TIC) and/or Recruitment Innovation Center (RIC)^3^ with the capacity and pertinent expertise (Figure 1). The assigned TIC or RIC team convenes an introductory call with the study investigator to ensure that initial requests for resources, such as single IRB, site expression of interest, electronic health records-based recruitment, and/or recruitment and retention planning and materials, are appropriate and to determine whether additional, nonrequested resources are advantageous. Domain experts with prior trial experience in relevant therapeutic, methodological, and population areas are then identified. Next, a kick-off call is scheduled with the investigator, identified experts, and applicable resource leads to discuss topics such as the scientific premise, expected outcomes, and recruitment and retention plan. Additional, topic-specific calls are scheduled as needed. A final wrap-up call summarizes the guidance given and finalizes TIN resource provision. Based on an assessment of the project’s needs and potential benefits from further support, the TIC/RIC team may provide a recommendations report, recommendations plus resources, or a comprehensive consultation. A summary of completed initial consultations is presented to a TIN governance committee—the proposal assessment team (PAT)—comprising leadership from each of the TICs, the RIC, and NCATS. The recommendation for a comprehensive consultation requires an active discussion and affirmative vote from the PAT to proceed. A planned publication will describe the Comprehensive Consultation process.

## Results

**Figure 2.**
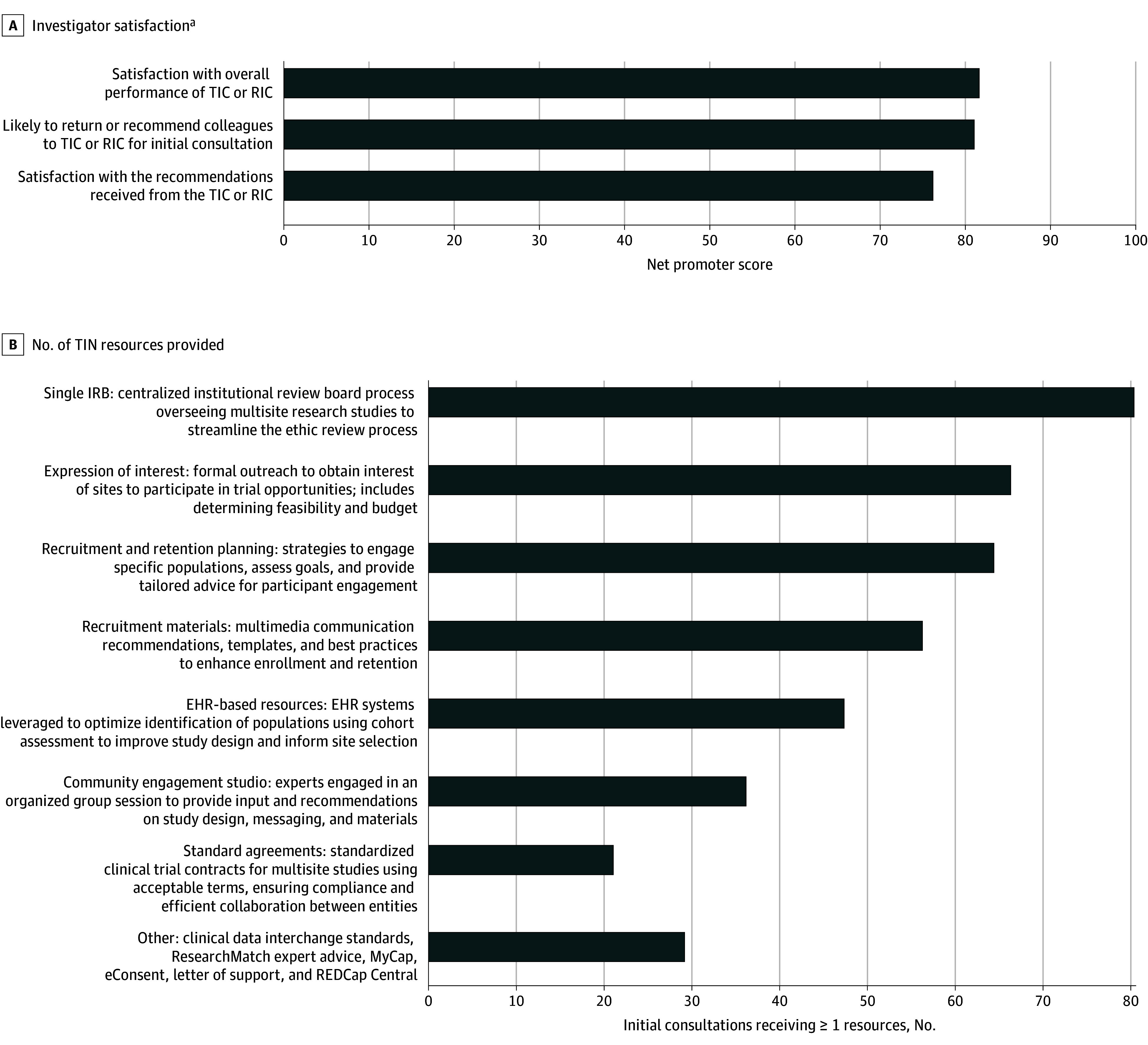
Trial Innovation Network (TIN) Initial Consultation Metrics IRB indicates institutional review board; RIC, Recruitment Innovation Center; TIC, Trial Innovation Center. ^a^Net promoter score (NPS) = % promoters − % detractors. Individual NPSs were averaged across all 3 questions for a total mean NPS. The NPS is interpreted as good (ie, any positive score above 0), excellent (ie, a score of 50-69), or world class (a score of ≥70). NPS scores are aggregated across all TICs/RIC and by individual TICs/RIC in instances where the principal investigator voluntarily self-identifies as part of the survey.

From October 26, 2016, until June 1, 2024, 445 proposals were submitted to the TIN for consultation, a median (IQR) of 46 (29) proposals per year. Thirteen inaugural proposals received in 2016 were developed as use cases to organize TIN processes, with 7 proposals supported as demonstration projects (54%) and 6 as pilot studies (46%). The TIN initial consultation process then started in earnest, and through June 1, 2024, a total of 432 proposals (97%) were assigned to a TIC/RIC for an initial consultation upon receipt, with all receiving a recommendations report. A total of 115 proposals (26.6%) received the recommendations report only, 189 requested and received targeted TIN resources (43.7%), 75 moved to a comprehensive consultation (17.4%), and 53 were either still active or on hold as of June 1, 2024 (12.3%) (Figure 2).

Starting in 2019, the TIN began sending clinical trial teams a 3-question satisfaction survey to provide feedback to the TIN, anonymously if desired (Figure 2). A net promoter score (NPS)^4^ is calculated across all completed survey responses for each question. Between 2019 and 2024, investigator ratings from 168 completed surveys resulted in an NPS rating of world class satisfaction with the TIN initial consultation process.

## Discussion

The TIN has built infrastructure and resources to help investigators improve and accelerate multisite clinical trials, starting with the initial consultation process. Over time, TIN leadership has identified classes of issues that impede timely completion of clinical trials, and developed innovations—tools, methods, and resources—to address them.^5,6^ US-based investigators who are proposing, planning, or conducting a multicenter study, with any type of funding or in any discipline, can request a consultation through the TIN website portal. Our assessment is limited by the absence of a comparable consultation network for benchmarking. TIN consultations are provided at no cost to investigators.

## Notes

Supplement.
Data Sharing Statement
