# Error in Author Affiliations

**DOI:** 10.1001/jamanetworkopen.2025.25918

**Published:** 2025-07-09

In the Research Letter titled “Enhancing Multicenter Trials With the Trial Innovation Network's Initial Consultation Process,”^1^ published May 29, 2025, there was an error in the author affiliation for Jeri Burr, MS, RN. Ms Burr is affiliated with the University of Utah Health, Salt Lake City, and not Johns Hopkins Institute for Clinical and Translational Research, Baltimore, Maryland. This article has been corrected.^1^

## References

[1] Harris PA, Wilkins CH, Lane K, . Enhancing multicenter trials with the trial innovation network's initial consultation process. JAMA Netw Open. 2025;8(5):e2512926. doi:10.1001/jamanetworkopen.2025.12926 40440017 PMC12123472

