# Supplementary material for: Enhancing Multicenter Trials With the Trial Innovation Network's Initial Consultation Process
Source: JAMA Netw Open. 2025 May 29;8(5):e2512926. doi: 10.1001/jamanetworkopen.2025.12926 (PMC12123472; doi:10.1001/jamanetworkopen.2025.12926)
Supplement: Supplement. — Data Sharing Statement [file jamanetwopen-e2512926-s001.pdf]

Data Sharing Statement

Harris. User Experiences of the Trial Innovation Network's Initial Consultation Process. *JAMA Netw Open*. Published May 29, 2025. doi:10.1001/jamanetworkopen.2025.12926

Data
Data available: No

Additional Information
Explanation for why data not available: No applicable data to share.
